# Assessing muscle function and oxidative profile in criollo horses participating in long loop rodeo before and after exercise

**DOI:** 10.29374/2527-2179.bjvm005823

**Published:** 2024-10-11

**Authors:** Andrielli Trentim Pereira, Ricardo Pozzobon, Bruno Leite dos Anjos, Leonardo Trentin Chaves, Érika Carla Smilgys, Ana Júlia Santos Thoma, Vinícius Leobet Lunkes, Juliana Sorraila de Oliveira, Cinthia Melazzo de Andrade, Júlio Cesar Mendes Soares

**Affiliations:** 1 Veterinarian, Programa de Pós-Graduação em Ciência Animal, Departamento de Clínica de Grandes Animais (DCGA), Hospital Veterinário Universitário (HUVet), Universidade Federal do Pampa (UNIPAMPA), Uruguaiana, RS, Brazil; 2 Veterinarian, DSc. DCGA, Hospital Universitário Veterinário HVU, Universidade Federal de Santa Maria (UFSM), Santa Maria, RS, Brazil; 3 Veterinarian, DSc. Departamento de Patologia Veterinária (DPV), HUVet, UNIPAMPA, Uruguaiana, RS, Brazil; 4 Undergraduate in Veterinary Medicine, HUVet, UNIPAMPA, Uruguaiana, RS, Brazil; 5 Undergraduate in Veterinary Medicine, HVU, UFSM, Santa Maria, RS, Brazil; 6 Veterinarian, MSc. Departamento de Patologia Clínica Veterinária (DPCV), HVU, UFSM, Santa Maria, RS, Brazil; 7 Veterinarian, DSc. DPCV, HVU, UFSM, Santa Maria, RS, Brazil; 8 Veterinarian, DSc. DCGA, UFSM, HVU, Santa Maria, RS, Brazil

**Keywords:** biochemical changes, long loop competition, equine, exercise, oxidative stress, alterações bioquímicas, competição de laço, equino, exercício, estresse oxidativo

## Abstract

Criollo breed is quite popular in Brazil, mainly in the Southern Region, and the long loop rodeo is one of the main sports modality adapted to this breed. The purpose of this study was to assess changes in the muscle and oxidative functions caused by exercises practiced in this sports modality. Data such as exercising frequency, covered distance, mean duration time and number of runs, and blood samples at the following times: before the tournament, on the final day of the event, and 18–24 h after the end of the tournament were collected. Were assessed lactate and the enzymes aspartate aminotransferase and creatine kinase (for the evaluation of the muscle function) and species reactive to oxygen, species reactive to thiobarbituric acid, catalase, superoxide dismutase and uric acid (for oxidative stress). Data were assessed through variance analysis associated with Tukey test (P ≤ 0.05), as well as through Spearman’s correlation (P ≤ 0.05). Significant creatine kinase and lactate increase in T1 associated with the maintenance of oxidative indicator levels in T1 and T18 reflected the significant muscle effort and the slight mobilization of oxidative parameters, which were compensated by the antioxidant defenses, since the assessed animals recovered after T18. There was no damage at cell level and the horses showed good muscle and antioxidant condition for exercising.

## Resumo

A raça Crioula é bastante difundida no Brasil, principalmente na região Sul, e o rodeio de laço comprido é uma das principais modalidades esportivas desta raça. O objetivo deste estudo foi avaliar as alterações da função muscular e oxidativas provocadas pelo exercício praticado neste tipo de prova. Para isso, foram coletados dados como frequência de treinamento, distância percorrida, tempo médio de duração e número de corridas e amostras de sangue nos tempos: 24 horas anteriores ao início da prova, após a última corrida do último dia de prova e 18 a 24 horas após o final da prova, em provas com duração de dois dias. Foram avaliadas as enzimas aspartato aminotransferase e creatinoquinase, lactato e o perfil oxidativo através de espécies reativas ao oxigênio, espécies reativas ao ácido tiobarbitúrico, catalase, superóxido dismutase e ácido úrico. Os dados foram estudados através de uma análise de variância associada ao teste de Tukey, considerando-se significativo para P ≤ 0,05, além de uma correlação de Spearman, considerando-se significativo P ≤ 0,05. As elevações significativas de CK e lactato em T1 associadas à manutenção dos níveis dos indicadores oxidativos em T1 e T18 refletiram um significativo esforço muscular e a discreta mobilização de parâmetros oxidativos que foi compensada pelas defesas antioxidantes, visto que os animais apresentaram-se recuperados após T18. Não houve danos a nível celular e os equinos mostraram uma condição muscular e antioxidante apta ao exercício.

## Introduction

Criollo horses are seen as animals of admirable rusticity and resistance (Vidart, 2004). Long loop rodeo, which is extremely important for the culture and economy of Rio Grande do Sul State, is not just one of the most commonly equestrian events practiced by this breed, but it also aims at simulating the work performed in the field at the time to lace and immobilize bovines for their management, at mean distance of 100 m, in fenced locations (Associação Brasileira de Criadores de Cavalos Crioulos, 2018). Based on results recorded for the same herein assessed animals in a different work conducted by our research team, it was possible concluding that high intensity and long duration exercises are performed in long loop events; they are performed in sand courts and do not count on a standardized training procedure. These animals also carry load weight higher than the ideal, but Criollo horses only presented physiological changes in the assessed clinical (heart and respiratory rates), hematological and biochemical analyses. The isolated or combined analyses applied to blood biochemistry can help identifying the risk of developing pathological changes caused by exercising (Fazio et al., 2014). All biological systems are in redox balance to actually balance the oxidative and reducing reactions in order to reach the proper conditions for life. Disturbances in redox homeostasis are caused by accumulation of oxidative molecules due to overproduction or loss of cell reduction ability (Gaschler & Stockwell, 2017). Exhaustive exercising can induce oxidative stress due to excessive oxygen consumption and to the high generation of reactive oxygen species (ROS) in the mitochondrial electron transport system. These species can have harmful effect on lipids, proteins and DNA, as well as have pro-inflammatory action (Deaton & Marlin, 2003).

Living organisms have antioxidant defense systems against ROS. It is possible saying that an organism is at oxidative stress when there is unbalance between ROS and the antioxidants, so that the first end up prevailing (Sies, 1986).

Exercising causes several changes in athlete equines in different sports modalities (Soares et al., 2011); however, Criollo horses, such as the ones participating in long loop rodeos, were not included in studies in this field. There is also missing data to assess the likelihood of these exercises to cause changes that could further reduce the animals’ athletic performance and even impair their well-being. Considering Criollo horses as the athletes they actually are, given their ability to participate in different equestrian events, mainly in the ones that resemble the work in the field, such as long loop lacing, the aims of the current study were to assess the muscle function and parameters indicative of oxidative stress caused by exercises imposed to Criollo horses in 2-day long loop rodeos, and to determine whether the type of exercises practiced by them can cause pathological changes in the assessed animals.

## Materials and methods

Inclusion criteria were that 49 animals from both sexes, at different age groups, should be registered in the genealogical record of the ABCCC, in order to standardize the profile of the animals, and to be participating in 2-day long loop rodeos. Data about exercising frequency, covered distance, mean time (run duration) and number of runs throughout the rodeo days were collected through a questionnaire applied at subscription time and at animals’ follow-up during the tournament. These data were numerically larger than those of animals who participated in the blood analyses; it was done in order to seek a more representative featuring of the profile of animals in the tournament.

In total, 10 mL of blood were collected from the jugular vein with the aid of sterile needle and syringe. It was deposited in tubes filled with sodium citrate to analyze the activity of catalase (CAT) and superoxide dismutase (SOD), as well as in tubes without anticoagulant to analyze creatine kinase (CK), aspartate aminotransferase (AST), lactate, uric acid, oxygen reactive species (ROS) and thiobarbituric acid reactive species (TBARS). Samples were collected at the following moments: 24 h before the beginning of the event (T0), after the last run in the last day of the event (T1) and 18 and 24 h after the end of the event (T18). The time interval of 6 h at T18 was determined due to the fact that equines who have finished the second day of the event, almost at the same time, were hosted in locations far from each other, and it impaired blood collection at the same time schedule.

28 horses were evaluated for biochemical indicators of muscle function, and they were analyzed in an automated equipment (BS-120, MINDRAY) with the aid of a commercial Kit. Plasma lactate was analyzed by the enzymatic UV method using lactate dehydrogenase. Serum CK and AST analyzes were performed using the kinetic method.

In total, 20 equines were assessed based on oxidative stress indicators.

Intracellular ROS production measurement was carried out by determining 2'-7'-dichlorofluorescein (DCF) as the indicator of reactive species production by cell components. Serum was obtained by centrifuging whole blood for five minutes at 1500 rotations per minute, and serum sample aliquots of 50 µl were added to medium with Tris-HCl buffer (10 mM; pH 7.4) and 2'-7'-dichlorofluorescein diacetate (DCFH-DA) (1 mM). After DCFH-DA addition, the medium was incubated in the dark for 1 hour until the moment of the procedure to measure fluorescence (stirring at 488 nm and emission at 525 nm – both adopted slit widths were 1.5 nm). Results were corrected based on protein content (Myhre et al., 2003).

TBARS levels were determined based on Jentzsch et al. (1996) by measuring malondialdehyde concentration (MDA) as product of lipid peroxidation due to reaction with thiobarbituric acid (TBA).

Briefly, the reaction mix had 200 µL of serum or of standard (0.3mM MDA), 1 mL of 0.2 M orthophosphoric acid - 250 µL of TBA (0.1 M) was heated to 95 °C for 45 minutes. Mean absorbance was 532 nm. TBARS serum levels were expressed in nmol MDA/mg protein.

The Nelson and Kiesow (1972) method was used for the CAT activity trial. The total blood sample was used in a reaction mix added with 50 mM potassium phosphate buffer (pH 7) and 10 mM hydrogen peroxide. The reaction rate of H2O2 was monitored at 240 nm for 2 min at room temperature. Enzymatic activity was expressed in nmol/mg protein/minute.

SOD activity was performed based on McCord and Fridovich (1969), with modifications. The trial was carried out at total volume of 1 mL added with 50 mmol of glycine buffer (pH 10), 60 mmol of epinephrine, and total collected blood with sodium citrate homogenized in phosphate buffer. Epinephrine was added to the solution and adrenochrome formation was recorded at 480 nm in ultraviolet-visible spectrophotometer (UV-VIS) for 5 min. One unit of SOD activity corresponds to the amount of enzyme required to inhibit oxidation in 50% epinephrine under experimental conditions. Results were expressed in U/mg of protein.

Uric acid values were found based on the rest of biochemical analyses carried out in automated analyzer (CM-200, WIENER LAB), by using the commercial kit, by the enzymatic colorimetric method.

Results were expressed as means; the variance analysis (ANOVA) one-way was performed in association with Tukey’s multiple comparison test, by taking into account significance at P ≤ 0.05.

Yet, collection time deltas were calculated in order to assess the intensity correlating some variables, namely: T1 mean values subtracted from T0 mean values (T1 – T0) and T18 mean values subtracted from T0 mean values (T18 – T0) to reduce variables’ distribution and to approximate them. Data such as number of runs and exercising were classified from 1 to 4, and from 0 to 3, respectively. Spearman’s correlation was carried out by taking into account significance at P ≤ 0.05. These results were expressed through dispersion graphics and tables.

## Results

The mean animal age was 7.2 ± 2.7 years; 36.7% (18/49) of males were neutered, whereas females comprised 63.2% (31/49) of the evaluated animals. The mean mass of animals reached 422.2 ± 35.8 kg. The body condition score results were distributed as follows: 3.0% of the animals scored 5 (1/33), 21.2% scored 6 (7/33), 60.7% scored 7 (20/33), and 15.1% scored 8 (5/33).

Most animals investigated in the current study (79.6% [39/49]) were bred under a semi-stable system, 20.4% (10/49) were free in the native field, 60% (06/10) were fed on native pasture, and their diet was supplemented with extra roughage (*Medicago sativa* hay) and concentrate. The roughage in the diet comprised native fields, *Medicago sativa* hay, *Avena sativa*, or *Lolium multiflorum* pasture, depending on the availability of the farm or breeder. The diet concentrate comprised commercial feed containing cereals, amino acids, vitamins, and minerals, 3,200 kcal/kg of digestible energy, and *A. sativa*, and *Zea mays* in the grains. The amount of concentrate supplied to the animals ranged 3–7 kg, on average, that is, from 0.7% to 1.6% of the live weight per animal, two to three daily. With respect to mineral supplementation, 30.6% (15/49) of the animals received common and mineralized salt *ad libitum*.

The evaluation of training management has shown that 69.4% (34/49) horses exercise from 1 to 2 times a week, 16.3% (08/49) of them do it from 3 to 4 times a week, 10.2% of the horses (05/49) exercise more than 4 times a week, and 4.0% (02/49) did not exercise not even once a week. Training sessions, although lacking a standardized frequency, comprised from 10 to 15 runs, on average, per training session. The sessions were conducted in similar tracks, or even in the same locations where the events were supposed to happen. The assessed animals performed 12.7 runs in 2-day rodeos, on average, which totaled mean covered distance of 3,658 ± 2,267 m. The mean speed of the evaluated horses was 6.4 ± 0.6 m/s.

**Figure 1:**
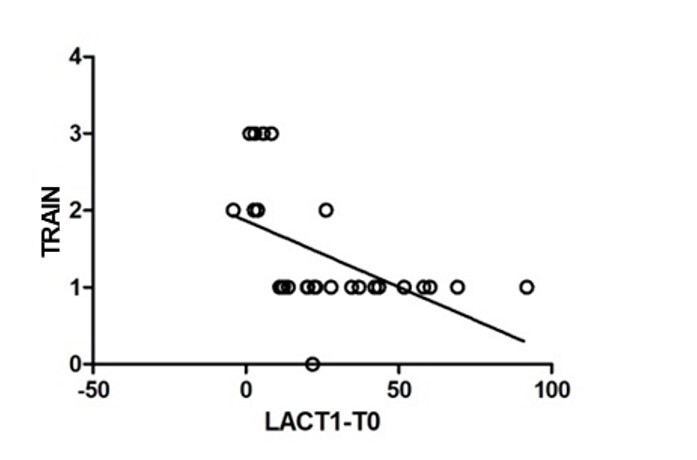
Graphic of the significant moderate and negative correlation of training frequency (0: without training, 1: once or twice a week, 2: three to four times a week, and 3: more than four times a week) and lactate delta (T1-T0) of Criollo horses participating in 2-day long loop rodeos. Animals who were subjected to less training presented higher lactate values.

There was a significant moderate negative correlation between T1-T0 lactate delta and animal training, where animals that trained less had higher lactate values (Figure 1).

Results of biochemical analyses, which made it possible observing significant increase (P ≤ 0.05) in CK and lactate, besides the non-changed AST, are shown in Table 1.

**Table 1:** Mean values, standard deviation (SD), maximum and minimum AST, CK and lactate in T0, T1 and T18 recorded for Criollo horses participating in the 2-day long loop events.

	**T0**	**T1**	**T18**
**CK (103-467 U/L)**			
Mean	416^b^	540.1^a^	487.6^ab^
SD	335.2	383.7	310.5
Min. and max. values	149-1,626	198-1,508	192-1,291
**AST (120-309 U/L)**	**T0**	**T1**	**T18**
Mean	342.3^a^	366.7^a^	346.4^a^
SD	270.2	207.0	187.3
Min. and max. values	170-1,454	176-1,161	206-1,062
**Lactate (10-16 mg/dL)**	**T0**	**T1**	**T18**
Mean	7.19^b^	33.03^a^	7.31^b^
SD	2.77	22.46	2.29
Min. and max. values	4.33-15.13	10.08-97.06	3.71-15.13

Different letters on the same line point out significant differences at P≤0.05. CK: creatine kinase; AST: aspartate aminotransferase.

Results recorded for oxidative stress indicators are presented in Table 2 – there was no significant changes among the three collection times.

**Table 2:** Mean values, standard deviation (SD), maximum and minimum ROS, TBARS, CAT, SOD and uric acid in T0, T1 and T18 recorded for Criollo horses participating in 2-day long loop rodeos, without significant change at any of the assessed times.

	**T0**	**T1**	**T18**
**TBARS (nmol MDA/mg prot)**			
Mean	8.97^a^	10.08^a^	9.70^a^
SD	3.04	2.23	3.13
Min. and Max. values	4.92-19.73	5.34-13.56	5.77-17.4
**ROS (DCFH-DA fluorescence)**	**T0**	**T1**	**T18**
Mean	37.79^a^	39.44^a^	41.24^a^
SD	4.87	5.27	5.23
Min. and max. values	30.67-50.25	28.01-50.1	31.54-52.77
**CAT (nmol/mg prot/min)**	**T0**	**T1**	**T18**
Mean	66.30^a^	66.22^a^	63.37^a^
SD	32.27	32.10	41.33
Min. and max. values	21.6-127.5	19.71-154.8	18.02-180.9
**SOD (U/mg prot)**	**T0**	**T1**	**T18**
Mean	14.36^a^	12.36^a^	12.57^a^
SD	4.03	3.51	3.06
Min. and max. values	9.65-27.21	7.47-17.98	6.78-16.76
**UA (mg/dL)**	**T0**	**T1**	**T18**
Mean	0.16^a^	0.12^a^	0.12^a^
SD	0.09	0.13	0.11
Min. and max. values	0.03-0.37	0.0-0.42	0.0-0.41

TBARS: species reactive to thiobarbituric acid; ROS: species reactive to oxygen; CAT: catalase; SOD: superoxide dismutase; UA: uric acid.

There were significant positive moderate to strong correlations if one takes into account the CAT, SOD and uric acid delta results, when they are correlated to covered distance, number of runs and mean time. Significant positive moderate correlations were observed by considering the CAT deltas when they are correlated to the lactate delta (T18 – T0) and significant moderate negative correlations were recorded when the TBARS delta results were correlated to CK and AST deltas (T18-T0) (Table 3).

**Table 3:** Significant correlations of (T1-T0) and (T18-T0) deltas of CAT, SOD and UA when covered distance, number of runs and mean time were correlated, significant correlations of (T1-T0) and (T18-T0) deltas of CAT when they were correlated to the (T18-T0) delta of lactate and significant correlations of (T1-T0) delta of TBARS when they were correlated to the (T1-T0) and (T18-T0) deltas of CK and to the (T18-T0) delta of AST.

	**CAT (T1-T0)**	**CAT (T18-T0)**	**SOD (T1-T0)**	**SOD (T18-T0)**	**UA (T1-T0)**	**UA (T18-T0)**	**TBARS (T1-T0)**
**CD**	0.742	0.697	0.719	0.641	NS	NS	NS
**Runs**	0.743	0.653	0.690	0.659	NS	NS	NS
**Mean time**	NS	NS	NS	NS	0.752	0.594	NS
**Lactate (T18-T0)**	0.461	0.478	NS	NS	NS	NS	NS
**CK (T1-T0)**	NS	NS	NS	NS	NS	NS	-0.516
**CK (T18-T0)**	NS	NS	NS	NS	NS	NS	-0.604
**AST (T18-T0)**	NS	NS	NS	NS	NS	NS	-0.509

CAT: catalase; SOD: superoxide dismutase; UA: uric acid; TBARS: species reactive to thiobarbituric acid; CD: covered distance; CK: creatine kinase; AST: aspartate aminotransferase; NS: non-significant.

## Discussion

Among enzymes whose serum must be determined in cases of muscle dysfunction, one finds AST and CK (Di Filippo et al., 2016). Reference CK (103-467 U/L) and AST (120-309 U/L) values recorded for Criollo horses in the age group 2-15 years have already been published (Franciscato et al., 2006).

CK results presented significant increase in T1; they were higher than the basal values in T18, but they were at downward trend in comparison to the time right after the end of the event. The recorded increase in CK is similar to values recorded 24h before and after the training session applied to Criollo horses in *Prova de Freio de Ouro*, which is another modality adopted for this breed (Da Cás et al., 2000). Significant AST elevations at different collection moments were not observed, but, based on Franciscato et al. (2006), values were slightly higher (342.3 ± 270.2) than those of the reference interval (Franciscato et al., 2006). However, they were within the reference interval (150 to 400 U/L) set for athlete equines, based on Rose and Hodgson (1994). By taking into consideration the differences between breeds and sports modalities, besides the great variability in reference values addressed in the literature, it is important highlighting the relevance of determining the reference values of muscle enzymatic activity determined for standard animals in comparison to the herein assessed ones, in this same modality.

The fact that the animals did not present significant AST increase is likely linked to the effects of exercising on their AST concentration, which depends on exercising intensity and duration, and on their health condition (Binda et al., 2016), since the assessed horses were healthy. Moreover, CK is more specific than AST in evaluations focusing on the presence of muscle injury (Gama et al., 2013). CK serum activity quickly increases after muscle injury compared to AST and presents maximum values within 6 to 12 h after the injury (Braz et al., 2016). If one takes into account the CK increase in T1, it is possible stating that 2-day rodeos were enough to cause changes in CK concentrations in the assessed animals. However, it is important considering that small-magnitude increase in enzymatic activity, as observed in most assessed animals, may result from the process to increase muscle cell membrane permeability, rather than from its rupture due to muscle injury (Braz et al., 2016), since – as shown in results observed in another study carried out by our research team, evaluating hematological and biochemical changes in the same animals, caused by the exercises praticed.

It was possible observing significant increase in lactate in T1, and this finding is indicative that horses competing in long loop rodeos perform exercises at anaerobic energy, because hypoxia is the main reason for increasing lactate production. Pyruvate catabolism should be the entrance of the Krebs cycle for lactate production (Landgraf Botteon, 2012). Since the concentrations returned to the basal levels in T18, it is possible suggesting a certain degree of horses’ adaptability to the type of physical effort, because the ability to metabolize the produced lactate points out the type of physical effort the horse is subjected to; it can also suggest the excess of effort, or not (Gama et al., 2013). The concentrations of blood lactate are regularly used to assess the ability level of athlete horses – V4 is the reliable quantitative variable of blood lactate to determine ability to the practiced exercise and competitive success (speed at which lactate reaches 4 mmol/L) (Soares et al., 2014).

It is possible visualizing such a statement through the significant negative moderate correlation between lactate delta T1-T0 and the training applied to the animals – animals who exercised less recorded higher lactate values (Figure 1). The anaerobic limit (V4) of Criollo horses ranges from 6 to 8 m/s (Amaral et al., 2013), and the mean speed of the evaluated horses was 6.4 ± 0.6 m/s - results recorded in a different article conducted by our research team.

Based on the present study, there was no significant difference in the ROS analysis, but there was slight increase in T1, and it kept on increasing from T18. ROS production can be harmful and have some damaging effects due to lipoperoxidation (Sies, 1986), such as oxidative injury in muscle fibers (Chiaradia et al., 1998). Hydroxyl radical is the most reactive one in biological systems. It has the shortest half-life; therefore, when it is produced *in vivo*, it damages the site close to the place where it was synthesized (Valko et al., 2007). If one takes into consideration the slight and non-significant increase in this parameter, and the isolated increase in the observed CK, it is possible suggesting that 2-day rodeos were likely enough to cause transitory increase in membrane permeability in muscle cells in the assessed horses. However, although the standard deviation in CK was high and some isolated animals presented values higher than the physiological ones (1291 U/L) – which remained 24h after the end of the exercise-, it is not possible not taking into consideration the possibility of punctual muscle damage and the likelihood of having some animals subjected to exercise overload. It is also related to other data in the current study, as well as to lack of training standards, mainly because of the fact that it is not an exclusively professional equestrian sports modality. Further studies with different and larger groups of Criollo animals are important to better elucidate the behavior of their muscle activity in long loop rodeos.

There was also no significant difference in TBARS levels, but it was possible observing slight increase in T1 - it was already at downward trend in T18. Malondialdehyde (MDA) is a secondary product from lipoperoxidation (Halliwell & Chirico, 1993); it is seen as biomarker of oxidative damage, since it can be freely measured based on TBARS (Vasconcelos et al., 2007). Lipid peroxides impose their toxic effect through two general mechanisms; extensive lipoperoxidation changes the assemblage, composition, structure and dynamics of lipid membranes. Lipid peroxides are also capable of spreading the additional ROS generation, or they degrade into reactive compounds capable of crosslinking DNA and proteins (Gaschler & Stockwell 2017).

Based on a study with jumping horses, TBARS’ plasmatic levels were significantly high right after the exercise, but they returned to basal levels 24h after the competition (Soares et al., 2011). Such a behavior shows that exercising stimulus leads to higher membrane lipoperoxidation indices, which is assessed based on the TBARS levels (Schneider & Oliveira, 2004). The slight increase, although non-significant, recorded in the present study confirms the use of this parameter to evaluate likely changes in membrane permeability.

Training reduces the TBARS levels after exercise tests in jumping horses compared to trained and non-trained animals (Andriichuk et al., 2016). By considering that the assessed rodeo Criollo horses did not have a controlled training program, so the light increase in TBARS levels after the exercise may have contributed to the mobilization for minimal and transitory oxidative stress.

The CAT activity in the assessed animals did not record significant differences at any evaluation time. Previous studies have shown that CAT is an enzymatic antioxidant that plays important role in protecting against oxidative stress during exercising (Andriichuk et al., 2016; Kolanjiappan et al., 2002). The present study did not show significant SOD differences in the assessed animals. SOD is an enzyme that catalyzes dismutation of the superoxide radical anion to hydrogen peroxide (H2O2) and oxygen (O2) (Vasconcelos et al., 2007). Studies have reported increase in the antioxidant ability of jumping horses, which was observed through SOD activity 24h after the exercising session, and it can be attributed to the compensatory response to the increased ROS production during exercising (Soares et al., 2011). Lack of increase in these enzymes once more pointed towards the likelihood of minimal oxidative and transitory stress caused by the practiced exercises, likely due to the fact that the exercise type was not enough to stimulate the mobilization of these enzymatic antioxidants, since TBARS and ROS did not present significant changes.

There were no significant changes in the uric acid levels in the assessed horses. Uric acid is an example of non-enzymatic endogenous antioxidant (Moffarts et al., 2004); it was featured as the first line of antioxidant defense in horses subjected to jumping competitions. It was observed after recording increased values in the post-event blood evaluations (Soares et al., 2011). Based on the present results, there was no mobilization of this antioxidant given the slightest possibility of oxidative stress caused by the 2-day rodeo.

The significant correlations among CAT, SOD, uric acid, TBARS, lactate, CK, AST, crossed distance, number of runs and mean time – animals recording the longer runs also recorded the greatest mobilization of these parameters – have shown the effectiveness of these important parameters to evaluate the muscle function and the oxidant status after exercising.

## Conclusions

The exercise session showed isolated CK increase, which returned to normal after rest; therefore, it is necessary having further studies to better assess the muscle conditions of these animals, a fact that can be related to animals’ lack of standardized training level. Anaerobic glycolysis was evidenced by lactate increase, which returned to basal levels after rest. If one takes into account the tested antioxidants and their significant correlations to the covered distance, number of runs and/or mean time, it is possible stating the positive response from their use to evaluate the animals’ oxidant status. The fact that significant increases in the oxidative profile parameters was not herein found is indicative of the animals’ good physical fitness. The horses did not suffer injuries at cell level when the performed muscle effort and the oxidative stress were assessed.
